# Leveraging biological replicates to improve analysis in ChIP-seq experiments

**DOI:** 10.5936/csbj.201401002

**Published:** 2014-01-31

**Authors:** Yajie Yang, Justin Fear, Jianhong Hu, Irina Haecker, Lei Zhou, Rolf Renne, David Bloom, Lauren M McIntyre

**Affiliations:** a Department of Molecular Genetics and Microbiology, University of Florida, Gainesville, Florida, USA; b UF Genetics Institute, University of Florida, Gainesville, Florida, USA; c Human Genome Sequencing Center, Baylor College of Medicine, Houston, Texas, USA; d Department of Applied Entomology, University of Giessen, Giessen, Germany; e UF Shands Cancer Center, University of Florida, Gainesville, Florida, USA

**Keywords:** ChIP-seq, peak identification, biological replicates

## Abstract

ChIP-seq experiments identify genome-wide profiles of DNA-binding molecules including transcription factors, enzymes and epigenetic marks. Biological replicates are critical for reliable site discovery and are required for the deposition of data in the ENCODE and modENCODE projects. While early reports suggested two replicates were sufficient, the widespread application of the technique has led to emerging consensus that the technique is noisy and that increasing replication may be worthwhile. Additional biological replicates also allow for quantitative assessment of differences between conditions. To date it has remained controversial about how to confirm peak identification and to determine signal strength across biological replicates, particularly when the number of replicates is greater than two. Using objective metrics, we evaluate the consistency of biological replicates in ChIP-seq experiments with more than two replicates. We compare several approaches for binding site determination, including two popular but disparate peak callers, CisGenome and MACS2. Here we propose read coverage as a quantitative measurement of signal strength for estimating sample concordance. Determining binding based on genomic features, such as promoters, is also examined. We find that increasing the number of biological replicates increases the reliability of peak identification. Critically, binding sites with strong biological evidence may be missed if researchers rely on only two biological replicates. When more than two replicates are performed, a simple majority rule (>50% of samples identify a peak) identifies peaks more reliably in all biological replicates than the absolute concordance of peak identification between any two replicates, further demonstrating the utility of increasing replicate numbers in ChIP-seq experiments.

## Introduction

The goal of chromatin immunoprecipitation (ChIP) experiments is to map the binding sites of a molecule (usually a protein) across the genome in a cell type or tissue [1]. ChIP assays start by cross-linking cellular interactions between DNA and the bound molecules with formaldehyde. The cross-linked chromatin is sheared into small fragments by sonication and the DNA-protein complexes of interest are recovered using specific antibodies, resulting in an enrichment of DNA fragments that were bound by the protein of interest. The cross-linking is then reversed and DNA fragments are released from the binding complex to be assayed. Usually there is a PCR amplification step to increase the amount of starting DNA. The first genome-wide ChIP studies used microarray (ChIP-chip) to analyze the DNA fragments [2, 3], which can now be sequenced directly (ChIP-seq) using massive parallel sequencing [4–6].

Different patterns of “peaks” will form at putative binding sites after the sequence reads are aligned to a reference genome. Peaks produced by site-specific binding of transcription factors are very narrow, while peaks of specific histone modifications are more diffusive and can cover large domains of DNA across several nucleosomes [7–9]. These two distinct types of binding are termed as point source and broad source, respectively. RNA polymerase II is an example of mixed source factors, which can form both highly localized and spreading peaks at different genome positions [10, 11].

In addition to sequences truly associated with the molecule of interest, random background noise is also present due to non-specific binding or biases in library construction and sequencing [12][13–16]. Peak placement depends upon the background in each independent experiment. The use of control samples may mitigate these biases but cannot eliminate all sources of noise. Replication is necessary to separate actual biological events from variability resulting from random chance [10, 18]. Technical replication measures a single biological sample repeatedly and allows estimation of the variability in the sequencing process. Biological replication measures multiple biological samples independently and enables inferences about the biological activity of the broader population where the samples are drawn. Biological replicates and their advantage over technical replicates have been well described in the context of gene expression studies such as microarrays (e.g. [19–22]) and mass spectrometry [23], and more recently in RNA-seq experiments [24, 25]. For ChIP-Seq experiments, with the ease of multiplexing and the plummeting costs of sequencing, increased sample sizes (i.e. number of replicates) are not only more affordable but are also becoming standard practice. For example, the ENCODE consortium requires a minimum of two biological replicates in ChIP experiments [26].

There is not yet consensus on how to analyze multiple-replicate ChIP-seq samples (Table 1). Pooling biological replicates is common in current protocols of ChIP-seq experiments. In some cases multiple biological samples were pooled and then divided into aliquots before sequencing [12]. Other investigators sequenced the biological replicates separately but pooled the sequencing data together before proceeding to data analysis [6, 18, 27, 28]. Pooling replicates is also integrated into the ENCODE framework [29], where the replicates were first analyzed separately to determine the Irreproducibility Discovery Rate (IDR) [30], and then pooled together for identification of the peaks passing the IDR.

**Table 1:** Approaches to analyze replicate ChIP-seq samples.

	Number of samples	Dependence on peak calling	Information from individual replicate	Examples
Pooling all replicates	No limitation	No limitation	Lost	[6, 12, 18, 27, 28]
IDR	Two	Optimized for peaks identified by SPP	Kept	[29, 30]
Select one best replicate	No limitation	No limitation	Lost	[42]
Majority rule	No limitation	No limitation	Kept	[65]

IDR combines pairs of replicates. However, IDR has many limitations. For the bivariate model of IDR, the preliminary peaks have to contain both high quality peaks and peaks that are most likely to be only noise, and the algorithm is currently implemented for only a few peak callers such as SPP [31] and MACS [32], with the caveat that the IDR developer has not optimized for MACS and recommends against it. However, investigators may prefer peak callers optimized for the binding factor of interest. The more stringent peak callers such as CisGenome [33] and QUEST [34] are not currently configured in the IDR package. Moreover, IDR relies on the ranking of the preliminary peaks and does not handle ties in the ranks, while such ties are common in ChIP-seq peaks. A true signal may be dropped by IDR when one replicate is noisier, because IDR chooses signals with consistent ranking over the signals that rank high in one replicate but low in the other. In this scenario, weak signals with consistent ranking between replicates are considered more credible than signals that were strong in one but weak in the other (inconsistent ranking).

In genomic experiments, independent processing of biological replicates is standard. Combined data may be unduly influenced by an outlier sample. Detection rates are also reduced, with binding sites with smaller signal-to-noise ratios being especially affected. However, detection is critical in ChIP-seq experiments for investigators who want to obtain maximal information. Another severe limitation of analyzing a single combined sample is that it precludes downstream quantitative comparisons across samples. Recently attention has been drawn to analyzing individual samples separately in ChIP-seq experiments [9, 35–41]. Some groups have proposed to focus on the analysis of one replicate, using the additional samples for confirmation only [42]. Others have compared overlapping peaks from biological replicates for transcription factor occupancy [41, 43], ChIP-seq quality control [44], and study of cell cycle phases [45]. Still, there is no consensus about how to leverage information provided by biological replicates.

In this study, we analyzed five ChIP-seq experiments with three or more replicates. Multiple methods for defining the consensus peaks using biological replicates were considered in order to minimize variability and maximize consistency. We confirm results from genomic studies and conclude that more than two biological replicates are essential for ChIP-seq experiments. We propose using a simple majority rule for peak identification and show that this yields more reliable peaks than absolute concordance with fewer replicates.

## Methods

### Data

We used five ChIP-seq data sets for this study. Two are previously unpublished and created in our labs. The raw data (fastq files) of the other three were downloaded from Gene Expression Omnibus (GEO).

- RNA Polymerase II ChIP-seq in *Drosophila melanogaster* with three replicates, and one input DNA control (GEO accession: GSE36107).
- Transcription factor NFKB ChIP-seq [46] (GEO accession: GSE19485) in human lymphoblastoid cell line GM10847. The cells were stimulated with TNF-α to activate NFKB regulation. This experiment consisted of five biological replicates and two IgG control samples.
- FOXA1 ChIP-seq in mouse liver with five biological replicates and three input control samples [47, 48] (GEO accession: GSE25836 and GSE33666).
- H3K4me3 ChIP-seq in *Drosophila melanogaster* with three biological replicates and three input control samples (unpublished).
- H3K27me3 ChIP-seq in mouse ganglia with three biological replicates, and no input control (unpublished)

### Analysis

**Figure 1:**
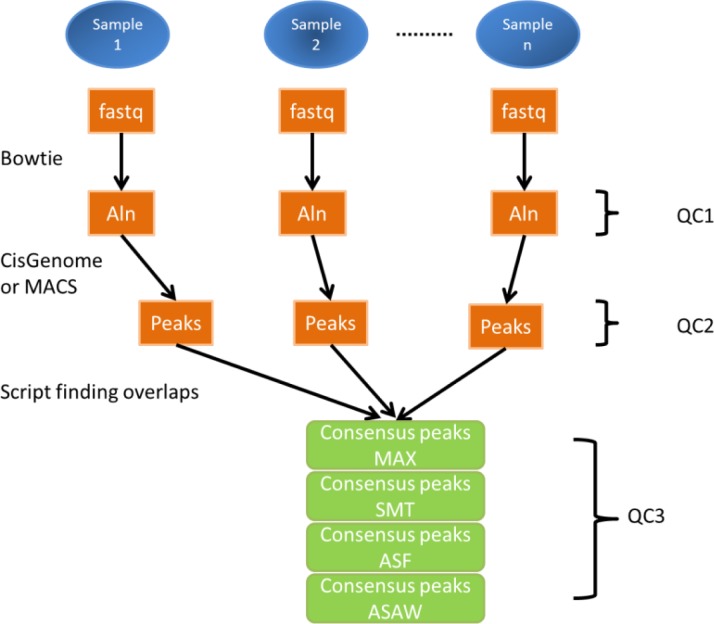
**Analysis pipeline for ChIP-seq experiments**. Each biological replicate is individually aligned to the appropriate reference (Aln), Peaks are identified (e.g. CisGenome or MACS). Quality control 1 (QC1) includes visual examination in a genome browser and quantification of total reads, uniquely mapped reads, and PCR bottleneck coefficient (PBC). Quality control 2 (QC2) includes evaluation of the number of peaks, the fraction of reads in peaks (FRIP), phantom peaks and common and unique peaks. Consensus peaks summarized from overlapping peaks with four different criteria (described in Methods and Figure 2). Quality Control 3 (QC3) examines correlation and agreement across replicates.

Biological replicates from each dataset were individually processed and underwent three levels of quality control (Figure 1). The fastq files were mapped to the genome (FlyBase 5.30 for drosophila, mm9 for mouse, and hg19 for human) using Bowtie [49] with options –m 1 –best –strata. Aligned reads were visualized in Integrative Genomics Viewer (Broad Institute) [50, 51] to check the overall read distribution shape and signal strength of the factor and the control at individual loci. Although not a quantitative metric, visible enrichment at known binding regions are expected in a successful ChIP-seq experiment. The PCR bottleneck coefficient (PBC) was calculated to measure approximate library complexity by taking the ratio of non-redundant uniquely mapped reads over all uniquely mapped reads. All the quality metrics based on the reads themselves and the initial alignments are QC1.

Peak identification from noisy ChIP-seq data is a challenging process, for which over 30 programs have been developed (for a review see [17]). In this study, we used two of the most popular peak callers, MACS2 [32] and CisGenome [33], which were found to perform better than other peak callers [12, 30]. These two algorithms are also representative of statistical models used for peak finding: MACS uses a dynamic Poisson distribution, while CisGenome uses a negative binomial distribution to account for the local biases across the genome.

Both programs were run with default settings with the input DNA samples as the control (except the H3K27me3 dataset for which the input control is unavailable). Notably, the default setting of MACS2 removes duplicate tags at the same location (–keep-dup=auto) and report peaks with FDR <0.05 (-q 0.05), while CisGenome does not automatically remove duplicates by default, and the cutoff for peak identification is a fold of enrichment >3 (-c=3.0) when a input control is used and >10 (-c=10) when the ChIP sample is analyzed alone.

Additional settings were explored. For the H3K27me3 data, we also present analysis results when removing duplicate tags first and using –c=6 besides those generated by the default setting. Parameter choices are important and investigators should spend time adjusting the parameters in order to obtain a reasonable list of binding sites for their factor of interest. Our intention here is not to compare the peak callers themselves but to use disparate peak callers with disparate settings and diverse data to see if there are universal conclusions about processing biological replicates that can be made.

QC2 is performed after peak identification and included summarizing the number of peaks identified as well as metrics to evaluate peak quality. The fraction of reads in peaks (FRIP, [33]) was calculated to estimate the global enrichment of signals against the background. Normalized strand cross-correlation (NSC) and relative strand cross-correlation (RSC) measure enrichment independently of peak calling. NSC is the normalized ratio between the fragment-length cross-correlation peak and the background cross-correlation. RSC is the ratio between the fragment-length peak and the read-length peak (http://genome.ucsc.edu/ENCODE/qualityMetrics.html).

For peaks independently identified from multiple replicates, it is unlikely that the exact peak position is the same across independent replicates. Peaks were considered overlapping among replicates if at least one nucleotide was shared. Unique and common peaks were identified across replicates. Peaks found only in a single replicate were considered unique. Peaks present in all replicates were considered to be common. The simple agreement coefficient was calculated as the number of overlapping peaks over all peaks identified in a pair of replicates. McNemar's test [56] evaluates the symmetry of identification for unique peaks, providing a measurement of agreement between replicates.

**Figure 2:**
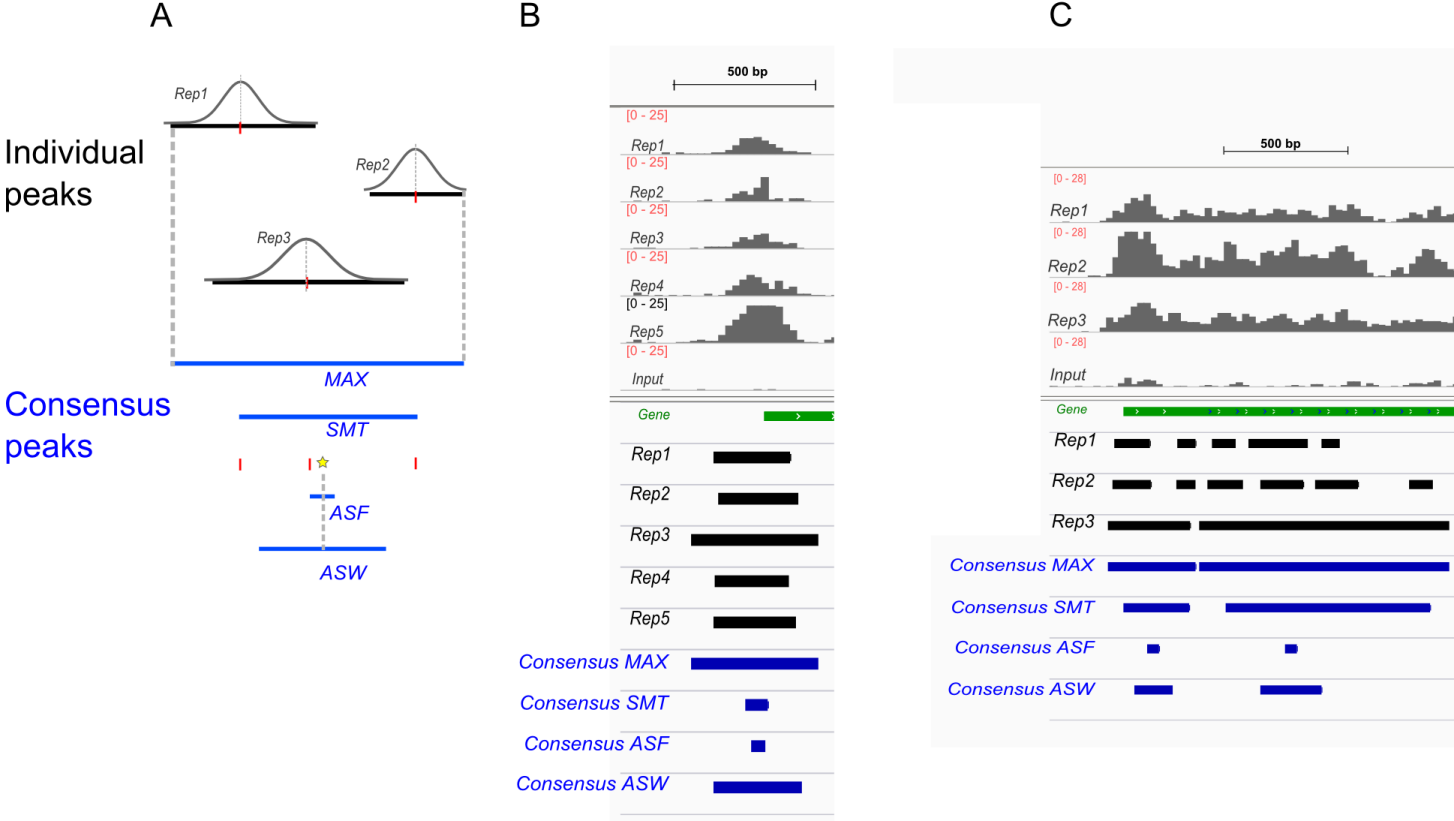
**Defining the consensus regions for overlapping peaks across replicates**. (A). Scheme showing different methods of combining individual peaks into a consensus. MAX: the maximum area encompassing all peak regions. SMT: the area between the summits of peaks. Summits of individual peaks are marked in red. The average summit of individual peaks is shown as the star. ASF: the area in the size of the footprint of the bound protein with the average summit as the center. ASW: the area centering the average summit in the size of the average peak width. (B) Snapshot of signals (grey bar charts on top), algorithmically identified peaks (black) and the consensus regions (blue) for point source factors that form narrow peaks at the transcription start site (TSS). The ChIP signals are distinct compared to the input control. The outlooks of the signals are highly similar for all five replicates when the signal range is not set but allows auto-adjustment to the local background (not shown). Here the range is set to a constant to allow comparison of the relative signal strengths, which vary across samples. The peaks identified in individual samples are similar in their position and width. (C) Snapshot for broad source factors whose binding signals span an entire gene (cropped at the 3’ end for readability). There are bigger differences in the identified peaks across replicates.

We explored several different ways to define a consensus region from peaks overlapping among a set of replicates with various exact positions (Figure 2). We compared: the maximum area encompassing identified peak regions (“MAX”); the area between the summits of overlapping peaks (“SMT”); the area encompassing the known footprint size for a specific binding molecule centered at the average summit (“ASF”), or using an empirical observation of average peak width to determine the boundaries again centered at the average summit (“ASW”). If peaks were identified only in a subset of replicates, the consensus peaks were determined from the subset where individual peaks had been identified. For each of these approaches, the coverage in consensus peaks was calculated as the Reads Per Kilobase per Million mapped reads (RPKM, [52]) for each sample. QC3 was developed to quantitatively evaluate the agreement across replicates. Consistency between pairs of replicates was explored using weighted Kappa coefficients [53] of ranked coverage (groups=5) and Spearman's correlation. Bland-Altman plots were also used to visually examine differences between the two replicates plotted against their mean [54, 55].

In many cases peaks were present in all replicates, but there are also cases where peaks were only identified in a subset of replicates. We proposed a “simple majority” rule and considered a peak identification to be consensus if it was detected in a majority of replicates, based on the reasoning that (1) if peak detection were random the likelihood of seeing a peak in the same location in multiple replicates would be small, and (2) given the noisy nature of ChIP-seq samples, a particular tool's chance of not identifying a peak in a region (false negative) is known to be large (Supplemental Figure 7). As the sample size of a ChIP-seq experiment increases, requiring an absolute consensus (100% agreement) will increase the false negative rate substantially. The majority rule allows for the simple extension of consensus between two replicates (the guideline proposed by [26]), to more complex situations. A majority consensus peak is supported by the majority of samples, allowing possible dissent in the other replicates. Naturally, this introduces the question of reliability of the peaks that have not been called unanimously. To determine whether the missing peak in some of the replicates was due to the lack of reads or merely a potential false negative from the peak discovery software, we tested for evidence that reads were enriched in the replicates where the software failed to identify them initially. For each sample, we used the peaks identified in that sample to estimate the distribution of RPKM values for peaks in that particular sample. RPKM values for peaks less than the 25^th^ percentile were considered the background. We used a Z-test where the null hypothesis is that its RPKM was not greater than the background. The peak was considered to be detected above background (DABG) when the null hypothesis was rejected (i.e. RPKM of the peak was greater than the 25^th^ percentile of the RPKM of all peaks of that sample).

The Gene Feature Format (GFF) file containing the genomic annotation of *D. melanogaster* was downloaded from: ftp://ftp.flybase.net/genomes/Drosophila_melanogaster/dmel_r5.30_FB2010_07/.

The promoters were defined as +/-2kb from the TSSs. The genic regions were taken as the upstream 2kb from the TSSs until the downstream 2kb from the transcript terminate sites (TTSs). Agreement between the RPKM of pairs of replicates was inspected using Bland-Altman plots for both promoters and genic regions.

## Results

### QC1 and QC2 show variability among biological replicates of ChIP-seq experiments

For all of the experiments we examined, the read level QC1 showed that the sequencing depth and quality varied among replicates (Supplemental Table 1 and 2). Sufficient numbers of total reads and uniquely mapped reads were necessary for binding site discovery. The RNAPII data met the rule of thumb promoted for the minimal mapped reads per sample, which is 2 million for drosophila, and 10 million for mammalian genome [26]. Under this rule the FOXA1 and NFKB experiments appeared to lack sequencing depth. The first replicate of the H3K4me3 data had much fewer reads compared to the other replicates. Consistent with their biological functions, the binding signals of RNAPII and H3K4me3 were associated with genic regions with more prominent peaks near the transcription start sites (TSSs) (Supplemental Figure 1). Clear and narrow peaks were found at the TSSs of known NFKB targets such as TP53 [57, 58], NFKBIA [59, 60], NFKB1 [61] (Supplemental Figure 1) and SHH [62].

QC2 revealed that the numbers of peaks independently identified were different for replicates of the same experiment (Supplemental Table 1) and the difference between peak calling programs was evident. The performance of same parameter settings depended upon the particular experiment, and there was not an immediately transparent mapping between the two underlying models of MACS2 and CisGenome. Using default settings, MACS2 [32] identified more peaks in the RNAPII data while CisGenome [33] identified more in other datasets. CisGenome peaks were also wider, especially for the NFKB data. Multiple consecutive peaks identified by MACS2 in RNAPII were frequently identified as a single peak by CisGenome (Supplemental Figure 1). The fraction of reads in peaks (FRIP) varied corresponding to the number of peaks being identified (Supplemental Table 1). Parameter exploration demonstrated the differences between MACS2 and CisGenome in the default settings beyond the underlying statistical models (Poisson vs. negative binomial). For example, the plentiful redundant reads in low PBC samples have to be removed deliberately for CisGenome but are automatically removed in MACS2. When this step was repressed in MACS2 by the --keep-dup option, the number of peaks became comparable to that identified by CisGenome for RNAPII and NFKB (data not shown). When redundant reads were removed, the number of peaks identified by CisGenome and the FRIP dropped noticeably and was closer to that of the default settings in MACS2 (Supplementary Table 3; Supplementary Table 4). Peak-independent measurements of enrichment such as Normalized strand cross-correlation (NSC) and relative strand cross-correlation (RSC) suggested three of the NFKB replicates were of medium quality, and the remaining samples were of high or very high quality (Supplemental Table 2).

### QC2: Proportion of common and unique peaks reflects the reproducibility of replicates

Without prohibitively costly independent validation experiments, the rate of false positive and false negative peaks cannot be accurately estimated. However, consistency of replicates provides a proxy for such an estimate, as the general assumption is that peaks identified in multiple samples, in approximately the same region, represent the same protein/DNA binding phenomenon. As showed by the peak level QC2, despite discrepancies in the number of peaks identified by CisGenome and MACS2 in individual replicates, the numbers of common peaks were more comparable between the two programs (Table 2; Supplemental Table 3).

**Table 2:** Numbers of common peaks. Common in all replicates: a peak was counted when it has overlapping peaks in each of the replicates. Common in the majority: a peak was counted when it has overlapping peaks in more than 50% of the replicates (i.e. three out of five, two out of three, etc).

	Program (using default settings)	Common in all replicates	Common in the majority of replicates
RNAPII	CisGenome	1,391	2,278
	MACS2	1,874	3,569
FOXA1	CisGenome	5	439
	MACS2	3	28
NFKB	CisGenome	113	432
	MACS2	62	781
H3K4me3	CisGenome	160	3,288
	MACS2	53	154
H3K27me3	CisGenome	29,989	80,284
	MACS2	7,709	17,039

The proportion of overlapping peaks between a pair of replicates reflects sample agreement, which was fair for the RNAPII and NFKB data (Supplemental Table 3a). The agreement was reasonable for the H3K27me3 data when MACS or adjusted CisGenome was used, but decreased when the peaks were identified using the default settings of CisGenome (Supplemental Table 3a). For H3K27me3 dataset, we focused on the results from adjusted instead of the default settings of CisGenome. Similarly, the default CisGenome also did not perform well for the H3K4me3 data. This was probably because CisGenome, unlike MACS2, was not optimized for histone signals (broad peaks). The FOXA1 data also had few reproducible peaks across replicates. Compared to the other datasets, the FOXA1 data appeared noisier in the genome browser and we were not able to observe noticeable peaks near known selected FOXA1 target genes. The metric we proposed (proportion of overlapping peaks) and the existing metrics (sequencing and mapped reads) all suggest high background noise in these data. The researchers in the original report combined the five replicates into one sample prior to analysis.

Generally, the number of peaks increases with the number of sequence reads for both CisGenome and MACS2 (Supplemental Table 1), consistent with previous studies [10]. McNemar's test [56] demonstrates that the unique peaks do not match for a given pair of replicates, with more peaks being identified in samples with greater sequencing depth (Supplemental Table 3b). However, this pattern was not strictly followed by the samples with high PCR bottleneck coefficient values (PBC>0.7).

### QC3: Consensus peaks and quantitative estimates of peak intensity

**Figure 3:**
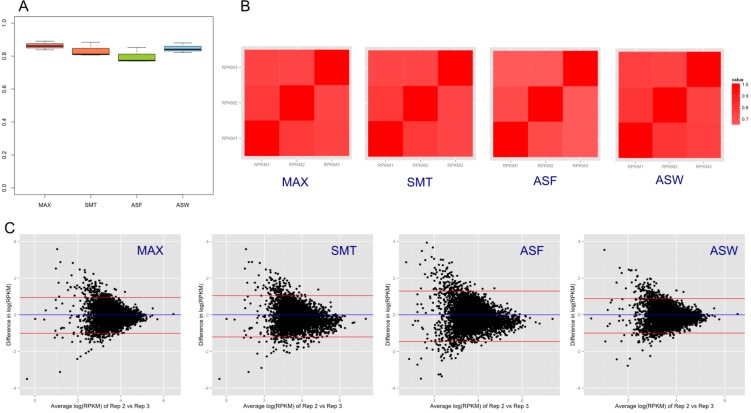
**Consistency across replicates of the RNAPII ChIP-seq experiment**. (A) Boxplot of weighted Kappa coefficients. The coverage in the consensus peak was binned into five ranked groups. The agreement of such ranked coverage between replicates was reflected by the weighted Kappa coefficients. A value over 0.75 indicates excellent agreement, which was met for all replicates regardless of the consensus being used. (B) Heat map of the Spearman correlation of the coverage in the consensus peak. Correlations were high. (C) Bland-Altman plots show the relationship between the difference (Y axis) and the mean (X axis) for a pair of replicates. Narrow and symmetrical plots reflect better agreement. Replicate 2 and replicate 3 are shown here, but other pairs (Replicate 1 *vs* Replicate 2, Replicate 1 *vs* Replicate 3) have similar patterns. Data shown are based on CisGenome peaks and more information is in Supplemental Figure 3.

Read coverage within specific peaks provides a quantitative measurement of enrichment above background. We calculated the Reads Per Kilobase per Million mapped reads (RPKM, [52]) in the consensus regions for common peaks (defined in Methods). Because differently defined consensus regions mostly varied in width (Figure 2), the choice of consensus region affected read coverage and in turn the estimate of sample agreement, though this effect was small (Figure 3; Supplemental Figure 2). ASF consensus peaks had relatively lower agreement across replicates, indicating that ASF is not a good choice of consensus despite its usage of biological knowledge of a protein's footprint size. It has been reported that although factors bind short regions of DNA (typically 5–25 bp), the DNA fragments that are pulled down typically cover a wider region of 150–600 bp around the binding site [13]. Therefore the width of identified peak regions does not always reflect the actual resolution of biological binding size. We also examined the enrichment in the corresponding regions of peaks identified in the replicate with the most reads. This is comparable with other ChIP-seq studies that arbitrarily selected one replicate as the reference sample (e.g. [42]). Unsurprisingly, such “consensus” peaks were heavily biased towards the sample that was selected as the standard (Supplemental Figure 2).

For RNAPII and NFKB, CisGenome called fewer peaks that had higher agreement across replicates (Supplemental Figure 3: BA plots with a narrower Y-axis where points are symmetrical around 0, higher Kappa and Spearman's coefficient), indicating these peaks were of higher quality. These peaks were also wider, including more reads that covered broader regions. In the H3K4me3 data, MACS2 identified fewer but higher quality peaks compared to CisGenome. The first replicate of H3K4me3 data was less correlated with the other replicates (Supplemental Figure 4), possibly an outlier, which was hinted by its lower read counts. The adjusted CisGenome and MACS2 yielded comparable Kappa and Spearman's coefficients for the H3K27me3 data. However, the distribution of the BA plots indicated that CisGenome peaks have better agreement (Supplemental Figure 6).

Despite the difference in the number of identified peaks, the RNAPII, NFKB and H3K27me3 replicates were highly correlated in terms of signal quantification (Figure 3; Supplemental Figure 5; Supplemental Figure 6). QC based on sequencing depth (QC1) and peak calling results (QC2) may identify the third replicate of NFKB experiment as failed; however, when measured quantitatively (QC3), it actually had good agreement with other samples (Supplemental Figure 5).

### Peaks identified in the majority of replicates are reliable

Due to the noisy nature of ChIP experiments and limitations of peak calling programs, peak identification varies across samples. Requiring support from all replicates for common peaks is likely to increase the false negative rate. We hypothesized that if a peak was identified in more than 50% of the replicates (i.e. two out of three, three out of five) there is sufficient support for its existence. More peaks were included as common under this majority rule (Table 2 “Common in the majority”). We tested whether the failure to identify a peak in some replicates is likely to be a false negative or whether there is no enrichment of binding in that area for that replicate. The probability of detection above background (DABG) was used to determine whether the observed signal in the putative peak region was greater than the first quartile of detected peaks in that sample (Z test p < 0.05, see Methods). Visual inspection using the genome browser found clear peaks at the TSS of known NFKB targets such as TP53 [57, 58], NFKBIA [59, 60], NFKB1 [61] and SHH [62], though these peaks were not identified in all replicates by CisGenome or MACS2 (Supplemental Figure 1). In addition, there were also distinct increases of signal near the TSS of BRCA2 and PTEN, both of which are known targets of NFKB [63, 64] but were not identified as peaks (Supplemental Figure 7). The absence of peaks identified at these regions may be the result of insufficient coverage or excessive noise at these genome positions.

**Figure 4:**
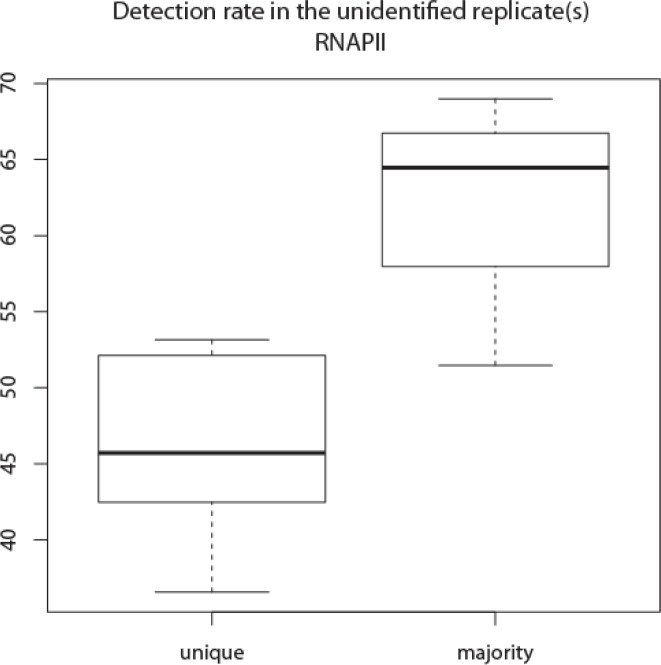
**Percentages of peaks detected above background (DABG) in replicates where no algorithmically identified peaks were present**. The read coverage (RPKM) in each identified peak, unique or common, was compared to the lower quartile of coverage in all peaks for that sample. The peak was detectable if the difference was statistically significant by a Z test. Peaks that were identified in the majority of replicates had a higher ratio to be confirmed by DAGB compared to those were unique in one replicate (Supplemental Table 3. The Y axis is the percentage of the peaks DABG and the mean is indicated by the sold line while the whiskers are the 25 and 75 percentile values.

Compared to the absolute consensus, more peaks were included as common under the majority rule (Table 2 “Common in the majority”). For the RNAPII data, peaks that were identified in the majority of replicates had a high confirmation rate using the test for detection above background (DABG) particularly when compared to tests for DABG for unique peaks, regardless the peak caller used or the consensus definition (Figure 4; Supplemental Table 4). Similarly in the H3K27me3 data, the DABG was 55% - 58% in the other replicates for the peaks identified solely in the third replicate, but increased to 81% - 85% when the peaks were also identified in an additional replicate. More than 92% of unique peaks in NFKB's first replicate were also supported by other replicates. This suggests that many genuine signals were missed by the peak callers. Consistent with the QC1 and QC2, peaks identified only in the third and fourth replicates of the NFKB data, were significantly above background only in 11% and 25% of the other replicates. When the majority rule was used, 100% of the peaks were also identified by DABG in the additional two replicates. DABG thus enables additional quality assessments, and an objective measure of whether peaks identified by the majority rule have supporting evidence in all replicates.

**Figure 5:**
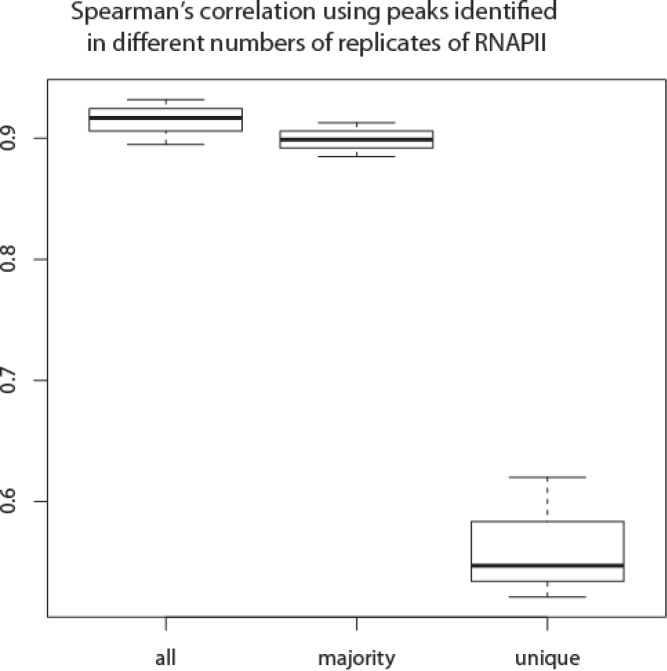
**Spearman correlation coefficients were similar when the peaks were identified in all replicates or in the majority of the replicates**. However, the correlation was much lower for uniquely identified peaks. The Y axis is the correlation coefficient and the mean is indicated by the sold line while the whiskers are the 25 and 75 percentile values.

Spearman's correlation between pairs of replicates was high, as expected, when using peaks that were identified by the peak callers in all replicates. The correlation was only slightly lower when the peaks that were identified in the majority were also included (Figure 5 showing RNAPII; Supplemental Table 6). However, when only one replicate was required for peak identification, the correlation in enrichment among replicates dropped dramatically (Figure 5 showing RNAPII; Supplemental Table 6), indicating that peaks identified in the majority of replicates were comparable to the common peaks, both of which were much more reliable than those identified in one replicate.

### Genomic features provide an alternative to algorithmically identified peaks

The performance of different methods for determining consensus peaks was dependent upon the mode of molecular binding, data quality and peak caller used. For the data we examined, MAX, SMT and ASW consensus peaks yielded a high estimate of consistency for point and mixed source factors. It was less conclusive for the broad source factors. Genomic features may serve as a reasonable alternative as quantification unit for well annotated genomes. For example, based on the biology that H3K4me3 marks are associated with TSSs, sample consistency can be inferred by inspecting the read coverage at TSSs. Even for factors whose functions are less defined, the regulation of many proteins are gene centric, therefore the binding strength in the nearby genic regions may provide a measure of the biological activity.

**Figure 6:**
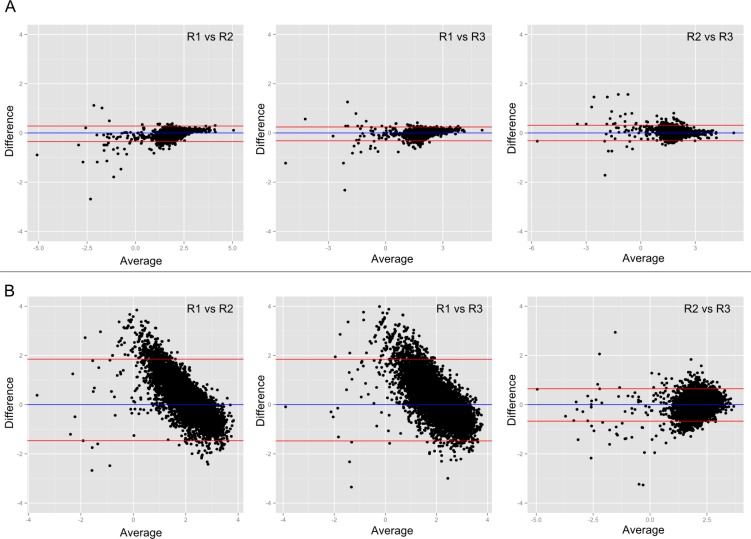
**Bland-Altman plots showing the sample agreement, using genomic features as the quantification unit**. The difference (Y axis) between a pair of replicates at the genomic feature (transcript for RNAPII [A] and TSS for H3K4me3 [B]) was plotted against the average of two samples. (A) Enrichment in the transcripts showed agreement for all replicates of the RNAPII data. (B) The first replicate of H3K4m3 appears to be an outlier sample, with little agreement with other replicates, while the second and third replicates agreed with each other in their enrichment near the TSS.

We calculated the coverage in the surrounding regions of TSS for the H3K4me3 data and coverage in the transcripts for the RNAPII data. Enrichment in the TSS surrounding regions was in good agreement for the second and third replicates of the H3K4me3 data (Figure 6). Consistent with other measures, the first replicate of H3K4me3 seems to be an outlier sample. The enrichment in the transcripts was in good agreement for all replicates of the RNAPII data (Figure 6).

## Discussion

Noise may be introduced during many steps of ChIP. Some may be technical issues in IP, library construction, or sequencing. Other noise may be due to biological differences among individual samples. As the tissue specificity of transcription factor binding and DNA modification has been demonstrated by the ENCODE project, we also expect that the tissue samples are more heterogeneous then the cell lines, which may be more heterogeneous than prokaryotes. The noise makes peak identification from ChIP-seq data a challenging task and demands some guidelines for considering all the sources of variability. Towards this end, we analyzed three publically available ChIP-seq data, and two of our own datasets with three or more biological replicates. Consistent with expression profiling techniques, we find that more replicates produce results that can be quantitatively as well as qualitatively evalauted. We propose that ChIP experiments should include at least three replicates and use the consensus peaks found in a majority of samples. Peaks common in all samples and peaks unique to a single sample can be used as an indicator of individual sample quality. Deeply sequenced experiments, such as the RNAPII data in this study, had better concordance among replicates than those with lower read counts. Encouragingly, reproducible peaks could still be determined from those studies with lower coverage.

Quantification of the signals in the consensus regions was consistent among replicates even when a peak was not initially identified for a particular replicate. Despite their distinct models for peak identification, the two different programs used in this study (CisGenome and MACS2) produced comparable quantitative measurements of consensus peaks and led to similar conclusions about the utility of replicates. Although we focused on default settings for this exercise, adjusting settings on peak callers can improve the concordance of peak identification among replicates.

The real binding sites are unknown for most ChIP studies. The strategy that requires identification of a peak in all replicates (absolute consensus) will exclude genuine binding sites. The failure to detect a peak in a particular sample may be due to low coverage or high background at a particular peak position, in combination with the uncertainty in peak calling algorithms. A practical approach to maximize site discovery is to increase the number of replicates. We showed that peaks that were identified in the majority of replicates were likely to be enriched above background in the replicates where the initial peak calling process had failed. When more than two replicates were examined, many peaks that would be considered unqiue in the pair of replicates were confirmed in an additional replicate. Peaks identified in the majority (>50%) of replicates were frequently confirmed in the missing replicates when they were specifically tested for detection among background, while the confirmation rate for unique peaks were much lower, suggesting these majority peaks were more likely to be true positives. Equally importantly, no single replicates were the source of most discrepancies and so the inclusion of more replicates improved the number and quality of peaks for all replicates. The majority rule may be applied to other IP-seq studies. Twice as many microRNA binding sites were identified from two out of three replicates than from all three replicates using high-throughput sequencing of RNA isolated by crosslinking immunoprecipitation (HITS-CLIP) technology [65].

Real target sites may not recur uniformly across replicates above background as defined by a particular peak discovery algorithm. Annotation-based approaches provide quantification that is independent of peak calling. They are complementary to peak identification for promoter/transcript-associated protein binding, or can be employed when peak calling is difficult. Notably, they cannot replace peak callers, as many binding sites would be missed, as it has been demonstrated by previous ChIP experiments that transcription factors, even transcription activators such as STAT1^6^ and E2F1 [66, 67], can bind in regions of the genome previously unknown, though the function of the binding remains unclear.

The decade-long debates on replication for microarray experiments [68] and more recently RNA-seq data [69] applies to the current discussion of ChIP-seq data. Not only is an increase in replication sensible from a statistical point of view, allowing a quantitative assessment of differences between groups, it enables identification of a higher number of reliable signals out of the noisy ChIP-seq data. The more variablity in the sample source, the more biological replicates will be necessary. More replicates provide a shield against undercalling, as a particular peak caller is unlikely to identify all peaks in all replicates. In cases where a certain peak is missing in one sample but present in other replicates, the signal in the missing sample can be estimated from other replicates and tested for detection above background in that replicate.
